# Extra virgin olive oil diet intervention improves insulin resistance and islet performance in diet-induced diabetes in mice

**DOI:** 10.1038/s41598-019-47904-z

**Published:** 2019-08-05

**Authors:** Enrique Jurado-Ruiz, Leticia Álvarez-Amor, Lourdes M. Varela, Genoveva Berná, María S. Parra-Camacho, María J. Oliveras-Lopez, Enrique Martínez-Force, Anabel Rojas, Abdelkrim Hmadcha, Bernat Soria, Franz Martín

**Affiliations:** 1 Andalusian Center of Molecular Biology and Regenerative Medicine-CABIMER, University Pablo Olavide-University of Seville-CSIC, Seville, Spain; 2 Biomedical Research Network on Diabetes and Related Metabolic Diseases-CIBERDEM, Instituto de Salud Carlos III, Madrid, Spain; 3 Department of Molecular Biology and Biochemistry Engineering, University Pablo de Olavide (UPO), Seville, Spain; 4 Instituto de la Grasa (CSIC), Campus Universitario Pablo de Olavide, Seville, Spain

**Keywords:** Type 2 diabetes, Obesity

## Abstract

Dietary composition plays an important role in the pathophysiology of type 2 diabetes. Monounsaturated fatty acid consumption has been positively associated with improved insulin sensitivity and β-cell function. We examined whether an extra virgin olive oil (EVOO) high fat diet (HFD) can improve glucose homeostasis. C57BL/6J mice were fed a standard diet or a lard-based HFD to induce type 2 diabetes. Then, HFD mice were fed with three different based HFD (lard, EVOO and EVOO rich in phenolic compounds) for 24 weeks. HFD-EVOO diets significantly improved glycemia, insulinemia, glucose tolerance, insulin sensitivity and insulin degradation. Moreover, EVOO diets reduced β-cell apoptosis, increased β-cell number and normalized islet glucose metabolism and glucose induced insulin secretion. No additional effects were observed by higher levels of phenolic compounds. Thus, EVOO intake regulated glucose homeostasis by improving insulin sensitivity and pancreatic β-cell function, in a type 2 diabetes HFD animal model.

## Introduction

The occurrence of metabolic diseases is rapidly increasing worldwide, and major risk factors for these diseases include being overweight or obese. Considering the increased incidence of these illnesses, the rise in type 2 diabetes (T2D) and nonalcoholic fatty liver disease (NAFLD) is particularly worrying. Lifestyle changes are responsible for this increase, especially a reduction in physical activity and chronic overnutrition^1,2^.

Dietary composition plays an important role in the pathophysiology of T2D and NAFLD. Dietary fats are of particular interest, with regard to not only the amount of fats but also the type of fats^3,4^.

In recent years, several studies have researched the influence of various dietary fats on T2D; however, their impact is still not fully understood. In addition, the underlying mechanisms of these fats are not clear. The proposed hypothesis is that dietary fats primarily modify the fatty acid composition of the cell membrane, thereby altering cellular functions related to insulin sensitivity^5^. Some studies have shown that feeding rats with different dietary fats affect endocrine pancreas structure and function^6,7^. Regarding pancreatic β-cells, the lipotoxicity hypothesis states that an excess of free fatty acids (FFAs) affects β-cell survival and insulin secretory functions^8^. Nevertheless, little is known about the effects of the different FFAs on β-cells.

Data from controlled intervention studies indicate that replacing saturated fatty acids (SFAs) and trans FFAs with monounsaturated fatty acids (MUFAs) and polyunsaturated fatty acids (PUFAs) reduces the risk of T2D^3^. In addition, a cross-sectional study of the general population in Spain revealed a favorable relationship of a MUFA-rich diet with β-cell secretion of insulin^9^. Most studies have assessed the effects of dietary fatty acids on the prevention of metabolic diseases; however, exploring the ability of certain FFAs to reverse problems in glucose homeostasis may be more clinically relevant.

One of the most important dietetic sources of MUFAs, particularly in the Mediterranean diet, is EVOO. In the case of NAFLD, it was recently shown that dietary EVOO modifies the FFA composition in the liver. This modification regulates genes and proteins involved in hepatic lipid metabolism, thereby rescuing HFD-induced steatosis^10^. Only a few studies have analyzed the impact of EVOO consumption on T2D risk factors, metabolic control and complications. In adult rats a long-term EVOO-enriched diet increase the immediate glucagon-like peptide response, although no improvement was observed on glucose tolerance or insulin response during an oral glucose tolerance test^11^. In the case of patients, the studies show that EVOO intake improves metabolic control^12,13^ and cardiovascular risk factors^14^ in T2D patients because EVOO lowers insulin resistance^15^. It is hypothesized that the effects of EVOO are primarily due to its MUFA and phenolic contents.

In this regard, little is known about why EVOO intake reduces insulin resistance or about the effects of EVOO on pancreatic β-cell function or if it is beneficial to increase the level of phenolic compounds that EVOO already have. We hypothesize that in addition to the amount of fat, the source of dietary fat or the presence of phenolic compounds also affects glucose homeostasis. We tested this hypothesis in a HFD mouse model fed EVOO. We found that EVOO intake regulated glucose homeostasis, improving insulin sensitivity and pancreatic β-cell function. In addition, the intake of an EVOO much richer in phenolic compounds did not increase the beneficial effects of EVOO on insulin sensitivity and β-cell function.

## Results

### EVOO-HFD-based diets improved BW, glucose homeostasis and insulin resistance

**Figure 1:**
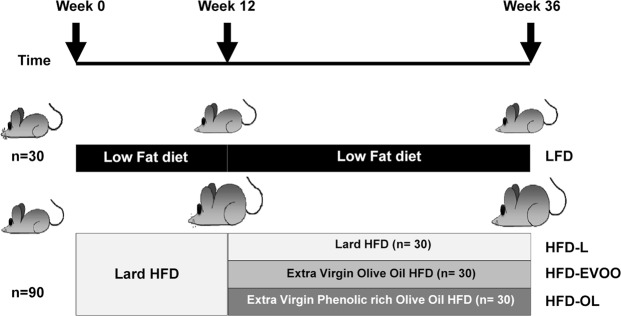
Experimental design. Five-week-old C57BL6J male mice were fed the control diet (LFD) and the lard HFD (HFD-L). After 12 weeks, the HFD-L group was divided into three groups and fed for another 24 weeks with (i) lard (HFD-L), (ii) EVOO (HFD-EVOO) and (iii) EVOO rich in phenolic compounds (HFD-OL). The LFD group was fed the control diet for the entire interventional study (36 weeks).

In Fig. 1, we can observe the experimental model where the animals are fed different diets during 36 weeks. The control group was fed with standard diet (LFD). The experimental group was fed with lard-HFD (HFD-L) during 12 weeks and then was split in three different groups: (i) mice fed with the same HFD-L; (ii) mice fed with EVOO (HFD-EVOO) and (iii) mice fed with an EVOO naturally much richer in phenolic compounds because its specific production process (HFD-OL). As illustrated in Table 1, the mean body weight (BW) of mice fed the HFD-L was significantly higher (*p* < 0.05) with respect to that of LFD group at week 12. At the end of the study, the BWs of the mice in the HFD-EVOO and HFD-OL groups were significantly lower (*p* < 0.05) than those of mice in the HFD-L group and not significantly different with respect to LFD group. In all cases, there were not significant differences within the amount of diet consumed by the animals (LFD: 4.1 ± 1.3 (n = 13); HFD-L: 5.0 ± 1.9 (n = 13); HFD-EVOO: 3.8 ± 1.6 (n = 18); HFD-OL: 5.4 ± 1.7 (n = 14) g/mouse/day).

**Table 1:** Mice body weight (g).

Week	LFD	HFD-L	HFD-EVOO	HFD-OL
0	29.5 ± 0.4	28.9 ± 0.1		
12	35.5 ± 0.5	45.4 ± 0.3*		
36	41.3 ± 0.8^a^	52.7 ± 0.9^b^	47.0 ± 1.4^a^	44.6 ± 1.5^a^

LFD: low fat diet; HFD-L: lard-based high fat diet; HFD-EVOO: EVOO-based HFD; HFD-OL: EVOO rich in phenolic compounds-based HFD. Values represent the means ± s.e.m; n = 30 for the LFD group and n = 90 for the HFD-L group until week 12; n = 13 for the LFD, n = 13 for the HFD-L, n = 18 for the HFD-EVOO and n = 14 for the HFD-OL groups at week 36. Student’s t test was used to evaluate differences between two means (LFD vs HFD-L, week 12; *p < 0.05) and de Kruskal-Wallis test for multiple comparisons (LFD, HFD-L, HFD-EVOO and HFD-OL, week 36). Different letters denote significant differences between groups; *p* < 0.05.

**Figure 2:**
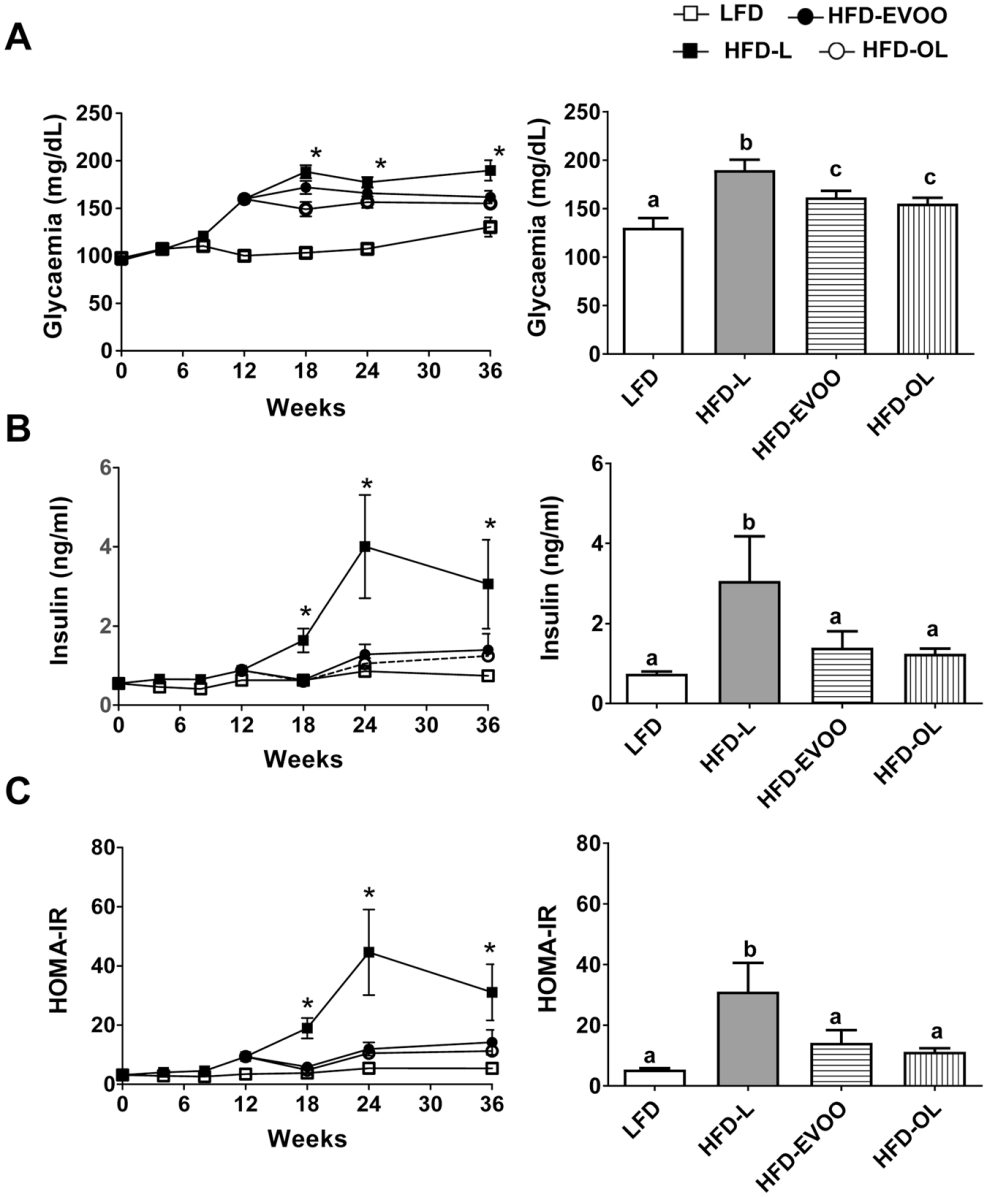
Effect of EVOO HFDs on glucose homeostasis and insulin resistance during the 36-week interventional study. Left panels show glycemia (**A**), insulinemia (**B**) and HOMA-IR (**C**). Right panels show glycemia (upper) insulinemia (middle) and HOMA-IR (low) at the end of the experiment. Values represent the means ± s.e.m. For LFD group n = 30 until week 12 and n = 13 since week 18 until week 36. For HFD-L group n = 39 until week 12. For the rest of the groups n = 13 since week 18 until week 36. Significant differences between groups were analyzed by one-way ANOVA followed by Bonferroni’s post-test. Different letters denote significant differences between groups (*p* < 0.05). *p < 0.05 LFD *vs*. the rest of the groups.

In the analysis of fasting glycemia (Fig. 2A) blood glucose levels were significantly increased (*p* < 0.05) in all HFD groups with respect to that of the LFD group after week 12. However, at the end of the interventional study, HFD-EVOO groups exhibited a significant decrease (*p* < 0.05) in glycemia compared with that in the HFD-L. Nevertheless, the glycemia of both HFD-EVOO groups, at the end of the study, was not significantly different with respect to LFD group. At week 18, the HFD-L group exhibited significantly increased (*p* < 0.05) fasting insulinemia levels with respect to the rest of the groups. Remarkably, both HFD-EVOO groups exhibited normalized insulinemia levels six weeks after the change from the HFD-L to the HFD-EVOO (Fig. 2B). The same phenomenon occurred with respect to HOMA insulin resistance (HOMA-IR) (Fig. 2C).

### EVOO-high fat-based diets improved glucose tolerance and insulin sensitivity

**Figure 3:**
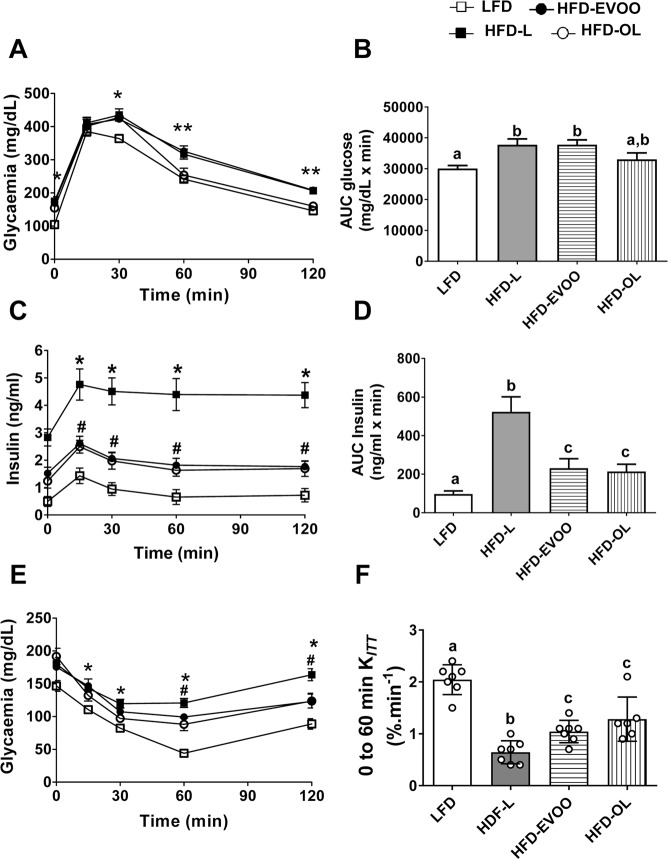
Effects of EVOO HFDs on glucose tolerance and insulin sensitivity at the end of the 36-week interventional study. IPGTT (**A**), AUC from IPGTT (**B**), Plasma insulin values from IPGTT (**C**), AUC from insulin values from IPGTT (**D**), ITT (**E**) and K_*ITT*_ (**F**). Values represent the means ± s.e.m. (n = 7). Significant differences between groups were analyzed by one-way ANOVA followed by Bonferroni’s post-test. *p < 0.01 between groups. In (**B,D**,**F**) different letters denote significant differences between groups (*p* < 0.01 for **B** and **F**; *p* < 0.001 for **D**).

Figure 3A shows that at the end of the interventional study, the HFD groups exhibited significantly higher (*p* < 0.05) blood glucose values 30 min after the glucose challenge. In addition, mice fed the HFD-L and HFD-EVOO exhibited the same AUC glucose values (Fig. 3B). However, from minute 60 to the end of the glucose challenge, the HFD-OL group exhibited the same glucose values as the LFD group. Moreover, HFD-OL-fed mice showed no significant difference in AUC glucose values when compared with LFD-fed mice, and in addition, there was a significant difference (*p* < 0.05) between the HFD-OL versus HFD-L and HFD-EVOO groups. This finding indicates that the HFD-OL, but not the HFD-EVOO, improved glucose intolerance. In contrast, insulinemia values from the IPGTT HFD-L group were significantly increased (*p* < 0.01) for plasma insulin during the entire test compared to those of the rest of the groups (Fig. 3C). In the case of the HFD-EVOO and HFD-OL groups, there were a significant increase (*p* < 0.05) when compared with the LFD group (Fig. 3C). AUC insulin values were significantly higher for the HFD-L group (*p* < 0.01) than for the other groups; however, the HFD-EVOOs group exhibited a lower increase in AUC insulin, although the increase was significant (*p* < 0.05), when compared with that of the LFD group (Fig. 3D). For the ITT test, the HFD-L group exhibited significantly higher glycemia values (*p* < 0.01) during the entire test compared to that of the LFD group (Fig. 3E). On the other hand, both HFD-EVOO groups showed a significant decrease (*p* < 0.05) in plasma glucose values from 60 min to 120 min when compared with those of the HFD-L group (Fig. 3E). The constant rate for glucose disappearance (*K*_ITT_) revealed significant insulin resistance in the HFD-L, which was significantly (*p* < 0.01) reversed in both HFD-EVOO groups, without no differences between the EVOO groups (Fig. 3F).

### EVOO-high fat-based diets restored IDE expression

**Figure 4:**
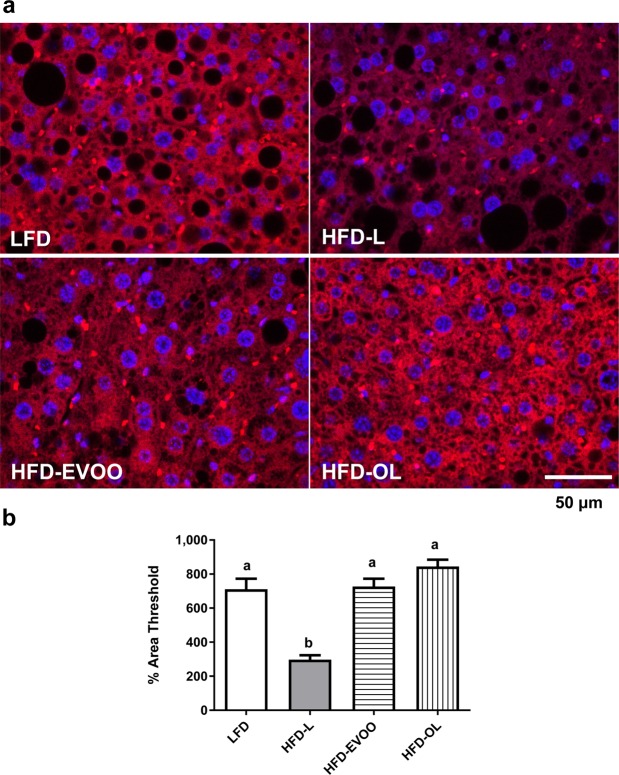
Effect of EVOO HFDs on liver IDE expression at the end of the 36-week interventional study. Representative fluorescence images showing IDE (red) (**a**). Nuclei were counterstained with DAPI (blue). Scale bar = 50 μm. Quantification of liver IDE expression as a percentage of the area threshold (**b**). Values represent the means ± s.e.m. (n = 5). Significant differences between groups were analyzed by one-way ANOVA followed by Bonferroni’s post-test. Different letters denote significant differences between groups (*p* < 0.001).

At the end of the interventional study, livers from the mice in the HFD-L group exhibited significantly decreased (*p* < 0.01) IDE expression (Fig. 4a,b) compared to those from mice in the other groups. In addition, livers from mice in the HFD-EVOO groups exhibited restored IDE expression compared to those from mice in the LFD group (Fig. 4a,b).

### EVOO-high fat-based diets inhibited apoptosis and increased β-cell numbers

**Figure 5:**
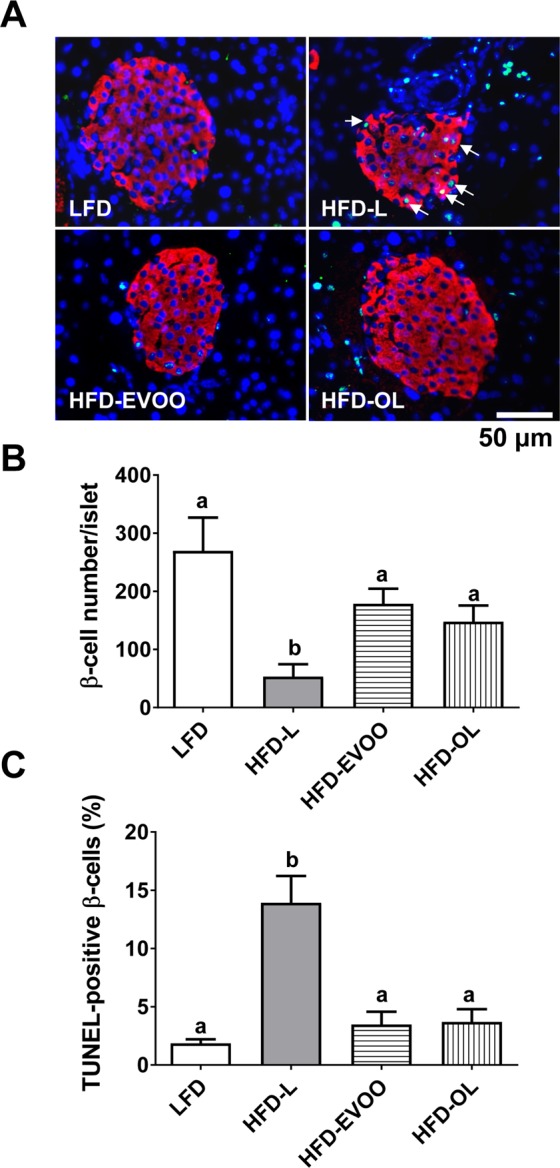
Effect of EVOO HFDs on β-cell number and apoptosis at the end of the 36-week interventional study. Representative confocal images showing insulin (red) and TUNEL-positive (green) cells (**A**). Nuclei were counterstained with DAPI (blue). Scale bar = 50 μm. Quantification of β-cell number per islet (n = 11) (**B**). Quantification of TUNEL-positive β-cells as a percentage per islet (n = 11) (**C**). Values represent the means ± s.e.m. Significant differences between groups were analyzed by Kruskal-Wallis test. Different letters denote significant differences between groups (*p* < 0.001) for panel b and (*p* < 0.01) for panel C.

At the end of the interventional study, islets from the mice in the HFD-L group exhibited significantly decreased numbers (*p* < 0.05) of β-cells (Fig. 5A,B) compared with those from mice the other groups, except HFD-OL group. In addition, islets from both HFD-EVOO groups exhibited restored numbers of β-cells compared with islets from the LFD group (Fig. 5A,B). Regarding β-cell apoptosis, the TUNEL assay revealed a significant increase in apoptosis (*p* < 0.01) in islets from the HFD-L group (Fig. 5A,C). In contrast, islets from both HFD-EVOO groups exhibited normalized apoptosis compared with islets from the LFD group (Fig. 5A,C).

### EVOO-high fat-based diets normalized glucose-stimulated insulin secretion (GSIS) and islet glucose metabolism

**Figure 6:**
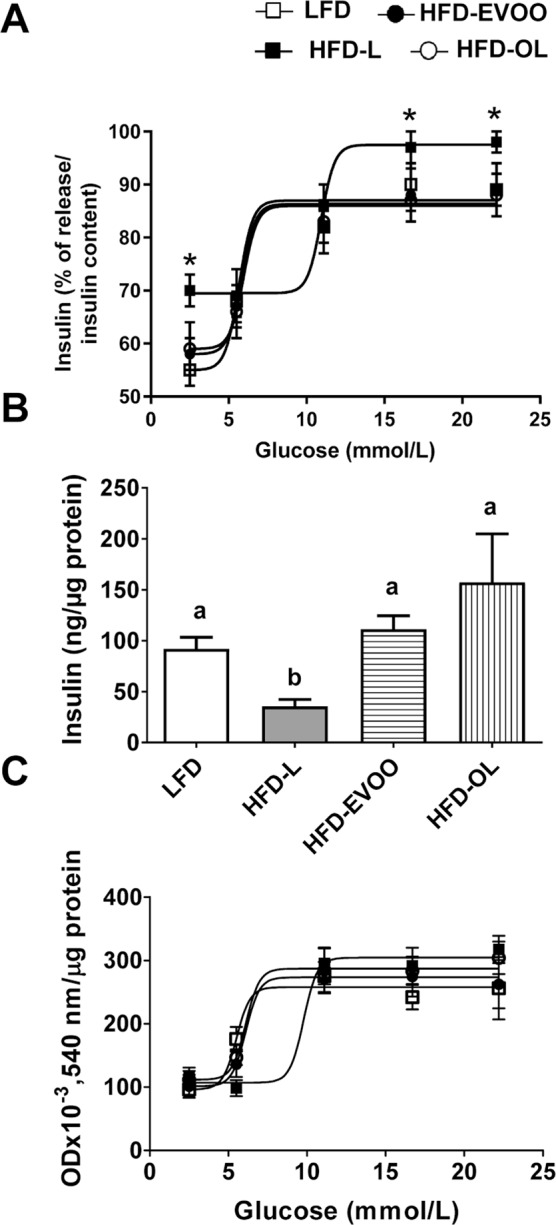
Effect of EVOOs HFD on glucose-induced insulin release and glucose metabolism at the end of the 36-week interventional study. *In vitro* glucose-induced insulin secretion (**A**), insulin content (**B**) and glucose-induced MTT reduction (**C**) from fresh islets obtained from the various intervention groups. Values represent the means ± s.e.m. (n = 7). In (**A**,**C)** significant differences between groups were analyzed by one-way ANOVA followed by Bonferroni’s post-test; *p < 0.05 between groups. In (**B)**, significant differences between groups were analyzed by Kruskal-Wallis test. Different letters denote significant differences between groups (*p* < 0.05).

The GSIS results from the islets from the various animal groups are shown in Fig. 6A. In all the groups, the relationship between insulin secretion and glucose concentration was sigmoidal. A broader glucose response range was seen in islets from the LFD and HFD-EVOO groups compared to those from the HFD-L group. Islets from the HFD-L group exhibited significantly increased (*p* < 0.05) insulin release at glucose concentrations higher than 16.7 mmol/L when compared with those from the rest of the groups. In addition, basal insulin secretion of this group was significantly higher (*p* < 0.05) with respect to that of the rest of the groups. Moreover, there exists a significant (*p* < 0.05) displacement to the right in the GSIS curve in islets from the HFD-L group (EC_50_ = 10.94 ± 0.11 mmol/L) compared with islets from the remaining groups (EC_50_ = 5.83 ± 0.15 mmol/L). Islets from the HFD-L group exhibited significantly decreased (*p* < 0.01) insulin content compared with the other groups (Fig. 6B). When glucose metabolism was analyzed based on MTT reduction, islets from all groups exhibited a sigmoidal increase in formazan production as a function of increasing glucose concentrations, reaching a maximal value of 11.1 mmol/L glucose (Fig. 6C). However, a significant (*p* < 0.05) displacement to the right in the dose-response curve was observed in islets from the HFD-L group (EC_50_ = 9.99 ± 0.19 mmol/L) compared with islets from the other groups (EC_50_ = 7.02 ± 0.11 mmol/L).

### EVOO-high fat-based diets improved insulin resistance and preserved β-cell function

**Figure 7:**
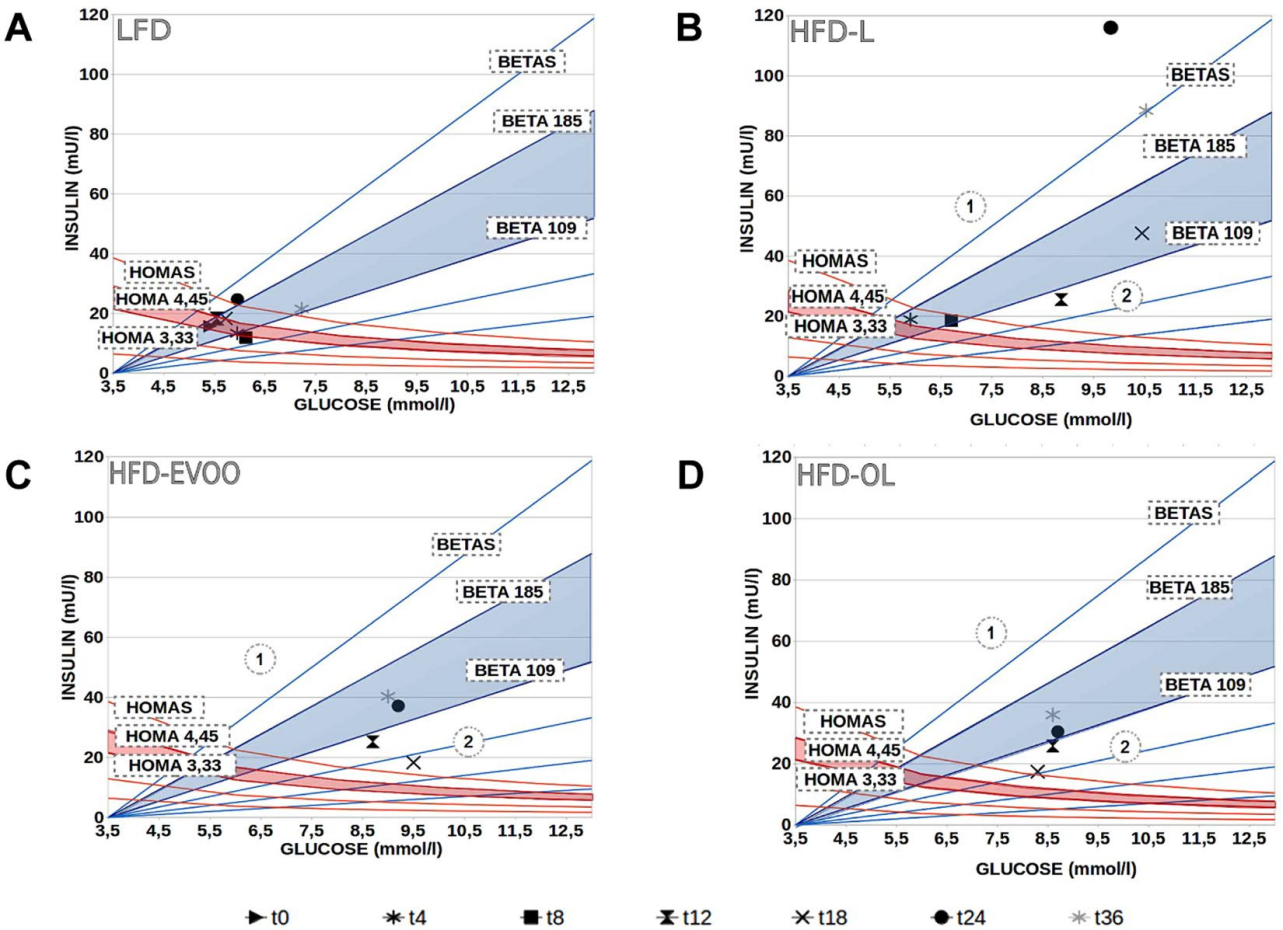
Effect of EVOO HFDs on insulin resistance, insulin sensitivity and β-cell function during the 36-week interventional study. Graphs representing fasting insulinemia *vs* glycemia with iso-HOMA-IR and iso-HOMA-%B curves at various weeks (t) of the study (0, 4, 8, 12, 18, 24 and 36). Blue shadow zones represent the normality range for HOMA-%B. Red shadow zones represent the normality range for HOMA-IR. The normality ranges were established using fasting insulinemia and glycemia values at week 0 (t0). The average value and the upper value of the confidence interval for the HOMA-IR index were used, as well as the average value and the lower value of the confidence interval for the HOMA-%B. Area 1 reflects increased HOMA-IR and HOMA-%B. Area 2 reflects increased HOMA-IR and decreased HOMA-%B. The fasting insulinemia and glycemia values represent the means ± s.e.m. of 30 mice for each nutritional intervention.

As shown on the graphs representing fasting insulinemia *vs* glycemia with iso-HOMA-IR and iso-HOMA-%B curves at various weeks of the study (Fig. 7), mice from the HFD-L group (Fig. 7B) were in area 1 after 24 weeks on the HFD-L diet. This result indicates that these mice exhibited insulin resistance, and the pancreatic β-cells released more insulin to compensate. However, mice from the HFD-EVOO groups after week 24 (Fig. 7C,D) exhibited lower insulin resistance, and therefore the values for these mice are within the blue shadow zone representing the normality range for HOMA-%B. This finding implies that the β-cells of these mice did not exhibit a compensatory increase in insulin release.

## Discussion

In this study, we examined the influence of the type of dietary fat in an animal model of HFD-induced T2D. In addition, we evaluated the importance of increasing the amount of phenolic compounds already present in traditional EVOO. Using a SFA-rich HFD we developed a T2D mouse model (Fig. 1). Table 1 shows that EVOO diet groups had a significantly lower BW compared to HFD-L mice, however there were not significant differences within the amount of diet consumed among the different groups. Similar results were found previously^10^. It could be possible, that high levels of EVOO intake had an effect on the energy balance physiology.

As shown in Fig. 2A, fasting blood glucose levels were less than 200 mg/dL, indicating that the mice had mild T2D suitable for the evaluation of maneuvers to regulate glucose homeostasis and metabolic control. Then, we studied the disease evolution when the dietary FFA source was replaced with MUFAs from EVOO. Our results showed that exchanging SFAs for EVOO in a T2D HFD mouse model improved glucose homeostasis, mainly throughout an increase in insulin sensitivity and a restoration of β-cell function. The latter effect is due to a reduction in apoptosis, an increase in β-cell mass and a normalization of glucose oxidation and GSIS.

The mouse model C57BL/6J is reported to be susceptible to HFD-induced obesity and T2D^16^. A few years ago, it was described that prior to the onset of T2D, HFD-induced obese female C57BL/6J mice develop β-cell functional adaptations as a compensatory response to obesity^17^. At the end of the manuscript, the authors suggest that managing these adaptations could be of interest in controlling glucose homeostasis and further regulating T2D progression. In this context, the composition of the diet in terms of the quality and quantity of fats may play an important role. Our hypothesis is that changes in fatty acid composition using EVOO may reverse the β-cell damage that occurs when β-cell adaptations fail, although the reduction of SFA intake should be considered as their toxic effects disappear. In spite of not being able to measure β-cell membrane fatty acid profile, we found a significant increase in oleic acid plasma levels in mice from both EVOO groups (Table S5). These data point out to our hypothesis.

We observed that an EVOO-based HFD significantly decreased fasting glycemia and normalized fasting insulinemia and insulin resistance at the end of the interventional study (Fig. 2).

It is important to note that the effects of EVOO and EVOO rich in phenolic compounds were only slightly different in terms of these parameters, as HFD-OL mice showed a slower fasting glycemia values, at the end of the study, when compared with HFD-L mice. Thus, it is likely that the observed effects of EVOO can be mostly attributed to the MUFAs. Another possible explanation is that both MUFAs and phenolic compounds are responsible of the observed effects, but once the phenolic compounds reach a certain level, larger quantities do not cause a really further improvement in certain metabolic parameters.

Our results are in disagreement with a previous study wherein SFA-HFD replacement for PUFA-HFD in C57BL6J mice reversed glucose intolerance more effectively than MUFA-HFD substitution^18^. A possible explanation for this discrepancy could be that in the aforementioned study, the replacement diet was administered for a shorter duration (4 weeks), whereas in our study, the change lasted for 24 weeks.

The IPGTT and ITT experiments (Fig. 3A,C,E) indicate that HFD-EVOO diets significantly improved the glycemic response and increased glucose sensitivity. The *K*_ITT_ values analysis revealed that both EVOOs counteracted the reduction in insulin sensitivity induced by the SFA-HFD. A previous study by Ryan *et al*.^19^ showed that replacing PUFAs with MUFAs (from an olive oil-rich diet) in T2D patients reduced insulin resistance. A possible explanation for this finding is that there were changes in the cell membrane FFA composition of insulin target tissues, with a higher proportion of oleic acid^10^. These changes could modify membrane fluidity and thereby induce conformational changes in the insulin receptors responsible for the observed increase in insulin sensitivity. The fact that mice from EVOO groups had significantly higher levels of plasma oleic acid could (Table S5) support this hypothesis. Moreover, in a previous report, we showed that mice fed with HFD-EVOO diets exhibited a significant increase in MUFAs, particularly oleic acid, in liver homogenates^10^. In addition, Santangelo *et al*.^11^ showed that consumption of polyphenol-rich EVOO, in T2D patients, improved their metabolic control. The authors propose that the intake of EVOO polyphenols modify circulating pro- and anti-inflammatory molecules. This finding suggests a possible role of phenolic compounds in the improvement of insulin sensitivity.

It is noteworthy, that EVOO-based HFD significantly decreased fasting glycemia, insulinemia and insulin resistance at the end of the interventional study (Fig. 2), as well as significantly lowered insulin release after a glucose challenge and significantly improved K_*ITT*_ (Fig. 3). However, only EVOO-HFD based in richer phenolic compounds (HFD-OL) had an impact on IPGTT, after 60 min (Fig. 3). We think that although the beneficial effects of EVOO account for an improvement in glucose homeostasis, their effects are not stronger enough to overcome a big glucose challenge. Nevertheless, when the levels of phenolic compound are increased (HFD-OL) a small improvement is reached, that makes possible to promote a recovery of the IPGTT response.

The regulation of glucose homeostasis depends on two factors: (i) peripheral insulin action and (ii) pancreatic β-cell insulin release. The first one is related to the action of insulin on peripheral tissues, and this issue has already been described. The second factor depends on pancreatic β-cell mass and function.

There exists a general consensus that SFAs are the most toxic to β-cells and that chronic exposure to SFAs induces apoptosis, particularly for *in vitro* cultures^20^. As shown in Fig. 5, the islets from the HFD-L group exhibited significantly increased rates of β-cell apoptosis and lower β-cell numbers. To date, the following factors have been implicated to explain this effect: (i) nitric oxide (NO); (ii) ceramide formation; (iii) inhibition of protein kinase B/Akt activity; (iv) activation of calpain-10; or (v) activation of protein kinase δ^21^. In contrast, pancreatic islets from mice fed the HFD-EVOO exhibited a decreased rate of β-cell apoptosis to control levels and normalized β-cell numbers (Fig. 5). In addition, the HFD-L also affects glucose metabolism, pancreatic islet insulin content and GSIS (Fig. 6). Glucose metabolism was assessed by the MTT reduction test, which enables the determination of β-cell metabolic activity^22,23^. Islets from mice in the HFD-L group showed a displacement to the right of the β-cell oxidative metabolism (Fig. 6C), with decreased glucose metabolism at glucose concentrations within 5.5 and 11.1 mmol/L. These data suggest that the metabolic steps of stimulus-secretion coupling are likely responsible for the decreased glucose sensitivity. As could be expected, data from GSIS test (Fig. 6A) were in agreement with the MTT observations. Thus, pancreatic islets from mice in the HFD-L group exhibited a decrease in GSIS sensitivity. Moreover, at basal and maximum stimulatory glucose concentrations, pancreatic islets from mice in the HFD-L group secreted more insulin. However, the fold increase of insulin secretion between basal and maximum stimulatory concentrations was lower in the HFD-L group than in the HFD-EVOO group and the control group. This finding indicates that in the pancreatic islets from mice in the HFD-L group, GSIS was impaired with respect to the rest of the groups. Notably, the changes observed in glucose metabolism, sensitivity and insulin content were reversed after 24 weeks in mice fed the EVOO HFDs (Fig. 6). In this case, the EVOO rich in phenolic compounds did not add additional benefits.

Concerning the normalization of apoptosis induced by the EVOOs, some *in vitro* studies performed in insulinoma cells^24^ and isolated rat islets^25,26^ suggest that MUFAs are well tolerated by β-cells. The hypotheses proposed to explain this phenomenon are as follows: (i) the aforementioned changes in membrane FFA composition (in this case, in pancreatic islets) modify membrane fluidity; (ii) MUFAs interact with either a cell-surface receptor or an intracellular receptor and (iii) ameliorate endoplasmic reticulum (ER) stress. In this case, several candidates have been proposed; however, none of these candidates is supported by strong evidence^21^. In addition, the effects of polyphenol compounds need to be taken into account. In this regard, it was shown that an olive oil polyphenol (tyrosol) exerts a protective effect against ER stress-induced β-cell death^27^.

To explain the normalization of glucose metabolism and sensitivity observed after the replacement of SFAs for MUFAs, we hypothesize that the recovery of β-cell mass is important. In addition, the recovery of glucose metabolism likely stems from an increase in glucose metabolic modulators of K_ATP_ channels, which are essential for maintaining proper GSIS. A recent study showed that in a C57BL6J mouse T2D model induced by a Western diet, the reversion to a standard diet restored the loss of β-cell functional mass^28^.

It is striking that mice in the HFD-L group, despite having much lower β-cell numbers (Fig. 5B), exhibited significantly higher plasma insulin levels in the presence of a glucose challenge (Fig. 3C). Insulin plasma levels represent a balance between insulin released by β-cells and the clearance of insulin^29^. Insulin clearance is primarily controlled by the IDE enzyme in the liver^30^. In addition, it has been demonstrated that cafeteria diet-induced obese mice presented a reduction in insulin clearance due to a downregulation of liver IDE expression^31^. As previously stated, mice fed the SFA-HFD exhibited liver damage (NAFLD), and the HFD-EVOO reversed this liver damage^10^. Thus, we investigated IDE expression in the liver of the different groups of mice. We found that the HFD-L group exhibited a significant reduction in IDE expression, which was normalized in the HFD-EVOO groups (Fig. 4). These data suggest that the reduction of IDE expression in the liver observed in the HFD-L group can explain the higher levels of plasma insulin levels found in this group.

Thus, our data show that EVOO intake can induce a recovery in β-cell mass and an improvement of β-cell function, in previously malfunctioning β-cells. This finding suggests that at the early stages of T2D, there exists a plasticity of β-cell functionality, which may have implications for intervention during the progression of T2D, as suggested by Gonzalez *et al*.^17^ six years ago.

The final figure (Fig. 7) allows us to evaluate the overall pathophysiological situation of mice fed with different diets along the various weeks of the nutritional intervention. In this evaluation, it is important to assess the insulin resistance (HOMA-IR) and the HOMA-%B, which indicate the β-cell secretory status and enable the prediction of the possible β-cell claudication. As shown in this figure, mice in the HFD-L group after week 12 exhibited insulin resistance, with the pancreatic β-cells releasing more insulin in an attempt to compensate, mixed with β-cell failure. Mice fed the HFD-EVOO exhibited lower insulin resistance and lower β-cell malfunction from weeks 12 to 18. From 24 weeks onward, β-cell function was recovered, as indicated by the values within the blue shadow zone, which represents the normality range for HOMA-%B. As previously indicated, our data show a discrepancy with the data published by Lamping *et al*.^18^, and our explanation is that the diet replacement period in that study was very short. The data shown in Fig. 7C,D indicate that the plasticity toward proper β-cell function takes several weeks following the damage.

In conclusion, the change from dietary fats present in a Western diet to those from EVOO improved glucose homeostasis in an animal model of diet-induced T2D. The effect was observed even in the presence of EVOO HFDs. EVOO diets significantly improved glycemia, insulinemia, glucose tolerance, insulin sensitivity and insulin degradation. Moreover, EVOO diets reduced β-cell apoptosis, increased β-cell number and normalized islet glucose metabolism and GSIS. In addition, the intake of an EVOO much richer in phenolic compounds increase the beneficial effects of EVOO on insulin sensitivity. Thus, EVOO intake regulated glucose homeostasis by improving insulin sensitivity and pancreatic β-cell function. It could be possible that part of the effects of EVOO intake would be related with the lower increase in BW observed in EVOO groups. Notably, the recovery of β-cell function took only three months. This finding suggests a notable plasticity of β-cells. Finally, we investigated the mechanisms underlying the known beneficial effects of EVOO consumption on impaired glucose regulation. More studies are warranted to determine these molecular mechanisms for a better understanding of the window of plasticity of β-cell function preceding the complete development of T2D.

## Methods

### Animals

Male C57BL6J mice (five weeks old) were purchased from Charles River (Cedex, France). Mice were bred and maintained at the Central Animal House of the Andalusian Center of Molecular Biology and Regenerative Medicine (CABIMER). All procedures with animals were approved by the Institutional Animal Care Committee of CABIMER (permission number 06-10-14-138) and performed according to the Spanish law on animal use RD 53/2013 and the European Community policy for Experimental Animal Studies (Directive 2010/63/EU). The mice were in groups of 5 animals per cage. All mice were allowed *ad libitum* access to the test diets and water throughout the 36-week research period. Fresh water and a fixed amount of feed were provided three times per week. BW, water intake (23.5 ± 3.2 ml/cage/day), food intake (20.9 ± 3.9 g/cage/day) and feed leftovers were recorded once per week throughout the study period.To determine these values four mice of each group were placed in the TSE Phenomaster monitoring system (TSE Systems GmbH, Bad Homburg, Germany). BW was recorded using a calibrated scale by transferring the mice to a clean empty weighing cage.

### Experimental design and dietary regimen

The mice were randomly separated into the following two groups for 12 weeks: a control (low fat diet, LFD) group (*n* = 30) and a lard-based HFD (HFD-L) group (*n* = 90) (Fig. 1). The LFD group was fed standard chow (caloric composition, 4% kcals from fat, 14.3% kcals from protein and 48% kcals from carbohydrate; 2.9 kcal/g total energy content; 2014 Teklad Global 14% Protein Rodent Maintenance Diet, Harlan, Spain; diet composition, Supplementary Table S1). The HFD-L group mice received 2014 Teklad chow modified with lard (caloric composition, 45% kcals from fat, 41% of these kcals from lard, 11% kcals from protein and 37% kcals from carbohydrate; 5.9 kcal/g total energy content) (Table S1). Next, the LFD group was fed 2014 Teklad chow for another 24 weeks, and the HFD group was randomly divided into three groups (HFD-L, HFD-EVOO, HFD-OL) for another 24 weeks (Fig. 1). The HFD-L group (*n* = 30) was fed as described for the initial HFD-L group. The HFD-EVOO group (*n* = 30) was fed 2014 Teklad chow modified with EVOO (caloric composition, 45% kcals from fat, 41% of these kcals from EVOO, 11% kcals from protein and 37% kcals from carbohydrate; 5.9 kcal/g total energy content; Table S1). The HFD-OL group (*n* = 30) was fed 2014 Teklad chow modified with EVOO rich in phenolic compounds (caloric composition, 45% kcals from fat, 41% of these kcals from EVOO rich in phenolic compounds, 11% kcals from protein and 37% kcals from carbohydrate; 5.9 kcal/g total energy content; Table S1). The fatty acid composition of the diets is provided in Supplementary Table S2. The phenolic fraction of both Picual EVOOs was isolated by solid phase extraction and analyzed by LC-DAD. The concentration of total phenolic compounds, expressed as tyrosol equivalents, was 104 mg/L for EVOO and 447 mg/L for EVOO rich in phenolic compounds (EVOO-OL). EVOO-OL was richer in phenolic compounds due to a specific production process; no phenolic compounds were added to the EVOO. The mean concentration of sterols, phenols and free fatty acids identified in the EVOOs used for this study are listed in Supplementary S3 and S4 Tables. The mean concentrations of fatty acids identified in the plasma of different mice groups are listed in Supplementary Table S5.

### Plasma collection and blood glucose

Animals were fasted overnight prior to blood collection for the glucose measurement. Blood was collected from the tail vein of conscious animals. For plasma isolation, approximately 900 μL of blood was collected into heparinized tubes by cardiac puncture. The blood was immediately processed by centrifugation for 15 min at 2500 × *g* at 4 °C, and then, the plasma was separated, aliquoted and frozen at −80 °C for further analysis. Blood glucose was measured using an automatic glucometer (Accu-Chek Aviva, Roche, Indianapolis, IN, USA).

### Islet isolation

Animals were sacrificed at the end of the dietary treatments by cervical dislocation in accordance with national guidelines. Islets of Langerhans were isolated by collagenase (Boehringer Mannheim, Mannheim, Germany) digestion as previously described^22^.

### Insulin measurements

Insulin measurements were performed as previously described^22^. Briefly, to measure glucose-stimulated insulin secretion (GSIS), the fresh collagenase-isolated islets were incubated for 1 h at 37 °C in fresh Krebs Ringer Bicarbonate Buffer (KRB) supplemented with 5.6 mmol/L glucose and 3% bovine serum albumin (BSA) (Sigma-Aldrich, St. Louis, MO, USA). The medium was continuously bubbled with a mixture of 95% O_2_:5% CO_2_ to obtain a final pH of 7.4. The medium was then replaced, and the islets were incubated in groups of 5 in 1 mL of KRB supplemented with 1% BSA and glucose at various concentrations (2.75, 5.5, 11.1, 16.7 and 22.2 mmol/L) for an additional 60 min at 37 °C. Then, the supernatant was collected and stored at −80 °C for the subsequent insulin measurements. In addition, 15-µL aliquots of the same samples were stored at −80 °C for protein analysis by Bradford assay. The insulin content was measured as previously described^22^. Insulin was assayed by ELISA using a kit from Mercodia (Mercodia AB, Uppsala, Sweden) per the manufacturer’s instructions. Standard curves and experimental points were performed in triplicate.

### Glucose and insulin tolerance tests

Intraperitoneal glucose (IPGTT) and insulin (ITT) tolerance tests IPGTT were performed at week 36 (at the end of the study period). For the IPGTT test, overnight fasted mice were injected intraperitoneally with glucose (2 g/kg body weight) and blood samples were collected at 0, 15, 30, 60 and 120 min. To measure glucose-stimulated insulin secretion, at same time points, blood samples were collected, and plasma was isolated and stored at −80 °C for the insulin analysis. For the ITT test, after 6 h of fasting, mice were injected intraperitoneally with 0.5 units/kg body weight human insulin (Humalin; Eli Lilly), and blood samples were collected at 0, 15, 30, 60 and 120 min. Blood glucose was measured using an automatic glucometer (Accu-Chek Aviva, Roche, Indianapolis, IN, USA). Insulin was assayed by ELISA using the Mercodia kit. Values for the IPGTT and ITT tests were calculated by estimation of the area under the curve (AUC) using the trapezoid rule^32^. Insulin resistance was calculated using the homeostasis model assessment (HOMA)^33^. The constant rate for glucose disappearance (*K*_ITT_) was calculated from the slope of the regression line obtained with log-transformed glucose values between 0 and 60 min after the administration of insulin (linear phase of glucose decay)^34^.

### MTT assay

Freshly isolated islets were incubated for 1 h at 37 °C in KRB supplemented with 5.6 mmol/L glucose and 3% BSA. The medium was continuously bubbled with a mixture of O_2_ (95%) and CO_2_ (5%) to obtain a final pH of 7.4. Subsequently, the reduction of C,N-diphenyl-N″−4,5-dimethyl thiazol 2-yl-tetrazolium bromide (MTT) (Sigma-Aldrich, St. Louis, MO, USA) was measured as previously described^22^. Briefly, batches of 15 islets were incubated in 1 mL of KRB supplemented with 1% BSA, 0.5 mg/ml MTT and glucose at various concentrations (2.75, 5.5, 11.1, 16.7 and 22.2 mmol/L) for 30 min at 37 °C. Then, formazan crystals were solubilized, the islets were sonicated and the optical density was recorded at 540 nm. The glucose concentration producing a response that was 50% of the maximum (EC50) was calculated as the mean negative logarithm (pD2).

### β-cell number and apoptosis

Purified islets were washed twice with KRB 5.6 mmol/L glucose and 3% BSA, fixed with 4% paraformaldehyde for 4 min in 0.1 mol/L PBS, washed with PBS and permeabilized with 0.02% Triton X-100 overnight. The primary antibody was an anti-insulin mouse monoclonal antibody (1:200 dilution; Sigma-Aldrich, St. Louis, MO, USA). The primary antibody localization was detected using anti-mouse TRITC (1:300 dilution; Sigma-Aldrich, St. Louis, MO, USA). Apoptosis was measured using a TUNEL staining *in situ* cell death kit (click-it Tunel Alexa Fluor 594 Imaging Assay; Invitro gen, Madrid, Spain). Proper controls for the secondary antibodies revealed no nonspecific staining. Nuclei were counterstained with 300 nM DAPI (Sigma Aldrich, Madrid, Spain). Confocal fluorescence images were captured with a Leica TCS SP5 confocal microscope (Leica, Wetzlar, Germany) at 2-µm intervals in the z-dimension. Images were analyzed using the open source image processing package FIJI. The β-cell number per islet was quantified by counting the number of cell nuclei within the insulin immunoreactive area. The apoptosis results are expressed as the number of TUNEL-positive β-cells over the total number of cells.

### Liver insulin degrading enzyme (IDE) expression

Livers were fixed in 4% paraformaldehydein PBS at 4 °C overnight and then processed for paraffin embedding with a Leica ASP200S tissue processor (Leica, Wetzlar, Germany). Sections with a thickness of 6 µm were cut on a Leica DM6000B microtome (Leica, Wetzlar, Germany) and processed for immunofluorescence analysis. A rabbit anti-IDE antibody diluted 1:2000 was used (Merck, Madrid, Spain, AB9210) as the primary antibody. The secondary antibody was anti-rabbit coupled to Alexa-568 (Thermo Fisher Scientific, Waltham, MA, USA). Nuclei counterstaining was performed with DAPI (1:500; Sigma Aldrich, Madrid, Spain). Fluorescence images were captured with a DFC 390 FX camera coupled to a Leica AF600 microscope (Leica, Wetzlar, Germany). Images were analyzed using the open source image processing package FIJI.

### Statistical analysis

The statistical analysis was performed using IBM SPSS Statistic Software Version 24. Statistical significance was analyzed byStudent’s t test for two means or one-way ANOVA with Bonferroni’s post-test for multiple comparisons, when the data comes from a normal distribution. We used Kruskal-Wallis test for multiple comparisons when data wasn’t normally distributed. Mean values followed by different letters were significantly different at *p* ≤ 0.05. All values were expressed as the mean ± s.e.m.

## Supplementary information

Supplementary information

## Data Availability

The data sets generated during and/or analysed during the current study are available from the corresponding author or reasonable request.
