# Supplementary material for: Extra virgin olive oil diet intervention improves insulin resistance and islet performance in diet-induced diabetes in mice
Source: Sci Rep. 2019 Aug 5;9:11311. doi: 10.1038/s41598-019-47904-z (PMC6683141; doi:10.1038/s41598-019-47904-z)
Supplement: Supplementary file 1 — Supplementary information [file 41598_2019_47904_MOESM1_ESM.docx]

**Extra virgin olive oil diet intervention improves insulin resistance and islet performance in diet-induced diabetes in mice**

Enrique Jurado-Ruiz^1¶^, Leticia Álvarez-Amor^1,2¶^, Lourdes M. Varela^1^, Genoveva Berná^1,2,3^, María S Parra-Camacho^4^, María J. Oliveras-Lopez^1,2,3^, Enrique Martinez-Force^4^, Anabel Rojas^1,2,3^, Abdelkrim Hmadcha^1,2^, Bernat Soria^1,2^, Franz Martín^1,2,3*^.

*^1^Andalusian Center of Molecular Biology and Regenerative Medicine-CABIMER, University Pablo Olavide-University of Seville-CSIC, Seville, Spain.*

*^2^Biomedical Research Network on Diabetes and Related Metabolic Diseases-CIBERDEM, Instituto de Salud Carlos III, Madrid, Spain.*

*^3^Department of Molecular Biology and Biochemistry Engineering, University Pablo de Olavide (UPO), Seville, Spain.*

*^4^Instituto de la Grasa (CSIC), Campus Universitario Pablo de Olavide, Seville, Spain.*

**SUPPLEMENTARY INFORMATION.**

**Supplemental material and methods indicating diets and EVOO characterization (sterols, phenols and free fatty acids analysis), as well as, analysis of mice plasma FFAs.**

*Sterols analysis*

Unsaponifiable lipids were determined as described by Abdallah *et al* (2015) saponifying 5 g of oil with 50 mL ethanolic KOH 12% mixed with 200 μL α-cholestanol (0.2% w/v), as internal standard. The mixture was heated at 60 ºC for 1.30 h. After cooling, 50 mL of H2O was added and the unsaponifiable matter was extracted four times with 50 mL petroleum ether. The combined ether extract was washed with 50 mL of ethanol-H_2_O (1:1). The ether extracted was dried over anhydrous Na_2_-SO_4_ and evaporated. The dry residues were dissolved in chloroform for TLC analysis. The unsaponifiable matter was separated into subfractions on preparative silica gel thin layer plates (silica gel 60 G F254), using 1-dimensional TLC with hexane/diethyl ether (65:35 by volume) as the developing solvent. The band corresponding to sterols was scraped off, extracted with chloroform/diethyl ether (1:1 v/v), filtered to remove the residual silica, dried in a rotary evaporator and stored at -10 ºC. An amount of 50 μL of solution was evaporated and derivatized with 50 μL of TMS derivatives (MSTFA/2-mercaptoetanol/NH4I) 1000:2:6 (v:w:v) and heated at 80 ºC for 20 min. After silylation the solution was directly injected to gas chromatograph. Sterols fraction was evaluated by GLC-FID using a HP5890 gas chromatograph, equipped with a FID, HP-5MS capillary column (30 m x 0.25 mm x 0.25 lm). The working temperatures of the chromatograph were 300 ºC at the injector, 260 ºC at the isothermal column, and 300 ºC at the detector. Quantification was made by the addition of internal standard (α-cholestanol for sterols fraction). Apparent β-sitosterol was calculated as the sum of β-sitosterol, Δ5,23-stigmasterol, clerosterol, sitostanol, and Δ5,24-stigmastadienol.

*Phenols analysis*

The phenolic fraction was isolated by solid phase extraction and analysed by reverse phase HPLC using a diode array UV detector according to Mateos *et al* (2001). The quantification of phenolic compounds other than flavones and ferulic acid was carried out at 280 nm using p-hydroxyphenylacetic acid as an internal standard, while flavones (luteolin and apigenin) and ferulic acid were quantified at 335 nm using o-coumaric acid as an internal standard. The results were expressed in mg kg^−1^.

*Fatty acid analysis*

EVOO and OL samples were transmethylated, and the resulting fatty acid methyl esters were analyzed by gas chromatography as previously described in Garces *et al* (1993).

*Plasma fatty acids analyses*

Lipids were extracted from approximately 100 µl of blood plasma using hexane-isopropanol as previously described (Hara and Radin, 1978). Total lipids, measured as fatty acids content and fatty acids composition were analyzed by GLC as previously described (Martínez-Force *et al.*, 2004). In brief, fatty acid methyl esters (FAMES) were obtained from isolated lipids by heating the samples at 80 °C for 1 h in 3 ml of methanol/toluene/H2SO4 (88:10:2 v/v). Heptadecanoic acid (30 µg) was added to each sample as an internal standard to allow quantification. After cooling, 1 ml of heptane was added and the samples were mixed. The FAMES were recovered from the upper phase and then separated and quantified using a Hewlett–Packard 5890A gas chromatograph (Palo Alto, CA, USA) with a Supelco SP-2380 capillary column of fused silica (30 m length, 0.25 mm i.d., 0.20 μm film thickness) (Bellefonte, PA, USA). Hydrogen was used as the carrier gas, with the linear gas rate being 28 cm/s. The detector and injector temperatures were set at 220 °C and the oven was set at 170 °C, with a split ratio was 1:50. Fatty acids were identified using standards (Sigma, St. Louis, MO, USA). TAGs were separated and quantified by GLC as previously described ([Fernandez-Moya et al., 2000](#_ENREF_13)) with an Agilent 6890 gas chromatograph (Palo Alto, CA, USA), and hydrogen was used as the carrier gas.

*References:*

1.- Abdallah, I.B. et al Content of carotenoids, tocopherols, sterols, triterpenic and aliphatic alcohols, and volatile compounds in six walnuts (*Juglans regia* L.) varieties. *Food Chemistry* **173**, 972-978, doi: 10.1016/j.foodchem.2014.10.095 (2015).

2.- Mateos, R et al. Determination of phenols, flavones, and lignans in virgin olive oil by solid-phase extraction and high-performance liquid chromatography with diode array ultraviolet detection. *J. Agric. Food Chem*. **49,** 2185–2192, (2001).

3.- Garcés, R. & Mancha, M. One-step lipid extraction and fatty acid methyl esters preparation from fresh plant tissues. *Anal. Biochem*. **211**, 139–143, doi: [10.1006/abio.1993.1244](https://doi.org/10.1006/abio.1993.1244) (1993).

4.- Hara, A., & Radin, N.S. Lipid extraction of tissues with a low-toxicity solvent. *Anal. Biochem*. **90**, 420–426, (1978).

5.- Martinez-Force, E., Ruiz-Lopez, N., & Garces, R. The determination of the asymmetrical stereochemical distribution of fatty acids in triacylglycerols. *Anal. Biochem*. **334**, 175–182, doi: 10.1016/j.ab.2004.07.019 (2004).

**S1 TABLE.**

Dietary composition of the experimental diets.

| **Diet (kcal %)** | **LFD** | **HFD** | **HFD-EVOO** | **HFD-OL** |
| --- | --- | --- | --- | --- |
| **Carbohydrate** | 48.0 | 37 | 37 | 37 |
| **Protein** | 14.3 | 11 | 11 | 11 |
| **Fat** | 4.0 | 45 | 45 | 45 |
| **% Saturated** | 0.6 | 18 | 5.1 | 5.1 |
| **% Monounsaturated** | 0.7 | 20 | 33.7 | 33.7 |
| **% Polyunsaturated** | 2.1 | 5.4 | 5.4 | 5.4 |
| **% from soybean oil** | 4.0 | 4.0 | 4.0 | 4.0 |
| **% from lard** | 0 | 41 | 0 | 0 |
| **% from EVOO** | 0 | 0 | 41 | 41 |
| **Energy (kcal/g)** | 2 | 4 | 4 | 4 |

All values in the table, with the exception of the fat source (soybean oil, lard or EVOO), represent the percentage of the total kcal in the respective diets derived from the indicated macronutrients. The values for the fat source represent the percentage of the total kcal from fat derived from the indicated source. LFD: low fat diet-control diet; HFD: high fat diet with the fat content consisting mainly on lard; HFD-EVOO: high fat diet with the fat content consisting primarily of extra virgin olive oil; HFD-OL: high fat diet with the fat content consisting primarily of extra virgin olive oil rich in phenolic compounds.

**S2 TABLE**

Fatty acid composition of the diets.

|  | **LFD** | **HFD-L** | **HFD-EVOO** | **HFD-OL** |
| --- | --- | --- | --- | --- |
|  |  | | | |
| **Fatty acids** |  |  |  |  |
| C16:0 Palmitic (%) | 0.5 | 5 | 1.6 | 2.1 |
| C18:0 Stearic (%) | 0.1 | 2.8 | 0.7 | 0.6 |
| C18:1n-9 Oleic (%) | 0.7 | 8.9 | 15.3 | 14.9 |
| C18:2n-6 Linoleic (%) | 2 | 3.3 | 3.3 | 3.1 |
| C18:3n-3 Linolenic (%) | 0.1 | 0.2 | 0.3 | 0.2 |

LFD: low fat diet-control diet; HFD-L: high fat diet with the fat content consisting mainly on lard; HFD-EVOO: high fat diet with the fat content consisting primarily of extra virgin olive oil; HFD-OL: high fat diet with the fat content consisting primarily of extra virgin olive oil rich in phenolic compounds.

**S3 TABLE**

Mean concentration of sterols and phenols identified in the EVOOs used for this study (mg/L). EVOO is extra virgin olive oil and OL is extra virgin olive oil richer in phenolic compounds.

|  | **EVOO** | **OL** |
| --- | --- | --- |
| **Sterols** |  |  |
| Cholesterol | 1.11 | 3.75 |
| Campesterol | 45.40 | 73.75 |
| Stigmasterol | 9.14 | 16.34 |
| Clerosterol | 15.25 | 23.16 |
| Beta-sitosterol | 1344.71 | 2177.23 |
| Sitosterol | 6.86 | 17.68 |
| Delta-5-avenasterol | 173.12 | 243.65 |
| Delta-5,24-stigmasterol | 10.47 | 11.82 |
| Delta-7-stimastanol | 4.43 | 6.00 |
| Delta7-avenasterol | 5.93 | 10.18 |
| Total sterols | 1616.42 | 2583.55 |
| **Phenols** |  |  |
| Hydroxytyrosol (HTY) | 7.50 | 239.52 |
| Tyrosol (TY) | 4.94 | 77.37 |
| 1^st^ HTY derivative | n.d. | 28.05 |
| 1^st^ TY derivative | 14.76 | 38.24 |
| 2^nd^ HTY derivative | 4.10 | 43.20 |
| 2^nd^ TY derivative | 12.36 | 18.52 |
| Vanillic acid | 1.63 | 1.30 |
| Vanillin | n.d. | 0.34 |
| HTY Acetate | 2.70 | 3.53 |
| TY Acetate | 15.08 | 14.07 |
| Pinoresinol | 8.16 | 4.34 |
| Cinnamic acid | n.d. | n.d. |
| Acetoxypinoresinol | 6.13 | 9.03 |
| Ferulic acid (mM/kg) | 9.19 | 7.83 |
| Total polyphenols | 86.55 | 485.35 |
| Total orto-phenols (mM/kg) | 14.30 | 314.30 |
| Total secoiridoids (mM/kg) | 31.22 | 128.00 |
| Total (ppm HTY/kg) | 104 | 477 |
|  |  |  |

**S4 TABLE**

Mean concentration of fatty acids identified in the EVOOs used for this study (g/100 g). EVOO is extra virgin olive oil and OL is extra virgin olive oil richer in phenolic compounds.

| **Fatty acid** | **EVOO** | **OL** |
| --- | --- | --- |
| C16:0 | 6.21 | 9.03 |
| C16:1n-9 | 0.18 | 0.12 |
| C16:1n-7 | 0.24 | 0.63 |
| C16:1n-5 | 0.16 | 0.12 |
| C18:0 | 3.27 | 2.96 |
| C18:1n-9 | 79.58 | 77.66 |
| C18:2n-6 | 8.91 | 8.08 |
| C18:3n-6 | 0.39 | 0.39 |
| C18:3n-3 | 0.95 | 0.91 |
| C20:2n-6 | 0.10 | 0.11 |

**S5 TABLE**

Mean concentrations of fatty acids identified in the plasma of different mice groups (mol%).

|  | **LFD** | **HFD-L** | **HFD-EVOO** | **HFD-OL** |
| --- | --- | --- | --- | --- |
|  |  | | | |
| **Fatty acids** |  |  |  |  |
| C14:0 | 3.62 | 3.00 | 2.99 | 3.13 |
| C16:0 | 32.34 | 25.34* | 28.31 | 26.47 |
| C16:1 | 1.54 | 0.87* | 1.27 | 1.24 |
| C18:0 | 18.46 | 21.05 | 19.32 | 20.37 |
| C18:1 | 16.86 | 14.88 | 24.56** | 26.23** |
| C18:1a | 1.81 | 1.00* | 2.23 | 2.01 |
| C18:2 | 14.23 | 17.30 | 12.33 | 12.21 |
| C20:0 | 0.50 | 0.36 | 0.71 | 0.86 |
| C20:1 | 0.33 | 0.31 | 0.44 | 0.37 |
| C20:2 | 0.71 | 0.78 | 0.82 | 0.69 |
| C20:3 | 0.89 | 0.90 | 0.95 | 0.71 |
| C20:4 | 7.78 | 12.38* | 5.28 | 4.98 |
| C22:6 | 0.94 | 1.84* | 0.79 | 0.73 |
| SAT | 55.85 | 51.59 | 51,33 | 50.83 |
| MUFAs | 20.54 | 17.06 | 28.50 | 29.85 |
| PUFAs | 23.61 | 31.34* | 20.17 | 19.32 |
|  |  |  |  |  |

LFD: mice fed standard chow; HFD-L: mice fed a high fat diet with the fat content consisting mainly on lard; HFD-EVOO: mice fed a high fat diet with the fat content consisting primarily of EVOO; HFD-OL: mice fed a high fat diet with the fat content consisting primarily of extra virgin olive oil rich in phenolic compounds. SAT: saturated fatty acids; MUFAs: monounsaturated fatty acids; PUFAs: polyunsaturated fatty acids. **p<*0.05 versus rest of the groups. ***p*<0.01 versus LFD and HFD-L groups.
